# Parametric Optimization of Thin-Walled 3D Beams with Perforation Based on Homogenization and Soft Computing

**DOI:** 10.3390/ma15072520

**Published:** 2022-03-29

**Authors:** Tomasz Gajewski, Natalia Staszak, Tomasz Garbowski

**Affiliations:** 1 Institute of Structural Analysis, Poznan University of Technology, Piotrowo 5, 60-965 Poznań, Poland; tomasz.gajewski@put.poznan.pl; 2 Doctoral School, Poznan University of Life Sciences, Wojska Polskiego 28, 60-637 Poznań, Poland; natalia.staszak@up.poznan.pl; 3 Department of Biosystems Engineering, Poznan University of Life Sciences, Wojska Polskiego 50, 60-627 Poznań, Poland

**Keywords:** homogenization, thin-walled structure, shell-to-beam, radial basis functions, artificial neural networks, parametrized optimization

## Abstract

The production of thin-walled beams with various cross-sections is increasingly automated and digitized. This allows producing complicated cross-section shapes with a very high precision. Thus, a new opportunity has appeared to optimize these types of products. The optimized parameters are not only the lengths of the individual sections of the cross section, but also the bending angles and openings along the beam length. The simultaneous maximization of the compressive, bending and shear stiffness as well as the minimization of the production cost or the weight of the element makes the problem a multi-criteria issue. The paper proposes a complete procedure for optimizing various open sections of thin-walled beam with different openings along its length. The procedure is based on the developed algorithms for traditional and soft computing optimization as well as the original numerical homogenization method. Although the work uses the finite element method (FEM), no computational stress analyses are required, i.e., solving the system of equations, except for building a full stiffness matrix of the optimized element. The shell-to-beam homogenization procedure used is based on equivalence strain energy between the full 3D representative volume element (RVE) and its beam representation. The proposed procedure allows for quick optimization of any open sections of thin-walled beams in a few simple steps. The procedure can be easily implemented in any development environment, for instance in MATLAB, as it was done in this paper.

## 1. Introduction

In recent years, thin-walled structures have gained more and more popularity, both as thin-walled beam profiles and trapezoidal sheets. They are most often used in constructions as load-bearing elements of structures, among others as roof bolts and purlins, spatial lattice trusses or in transom-column systems. In addition, thin-walled elements are used in the construction of facades and as bracings. In such applications, it is necessary to ensure the appropriate load-bearing capacity of the elements. Another application of thin-walled sections is their use for the production of architectural details. These include window and door leaves, balustrades and fences. Although they are not load-bearing elements, their weight and strength are still important and often optimized.

Generally, more and more often the aim is to obtain steel structures with the lowest possible weight while maintaining an appropriate load capacity. This can be achieved by using high-strength steel or by inserting appropriately spaced holes/perforations along the length of the element. Holes obviously reduce weight, but they can also act as mounting holes, which are used to attach a profile to another load-bearing element or, for example, to attach sandwich panels to such a profile, as was shown in [1]. These types of openings are typically placed at specific locations where connections are planned. Such an arrangement causes a local weakening of the cross-section and reduction of its stiffness. In turn, an arrangement of the mounting holes periodically along the length of the beam leads to a reduction in the stiffness of the cross-section along its entire length. However, such arrangement of the openings is advantageous as it allows the connection point to be freely adjusted on the construction site. In addition, the use of openings in thin-walled structures may be conditioned by the installation, such as fire sprinkler system [2] and electrical wiring. Due to the increasing development and emphasis on the creation of intelligent buildings, the number of installations will gradually increase, which will result in the need to use more openings in cross-section.

Designing load-bearing elements, including thin-walled purlins usually consists of selecting the appropriate cross-section from the manufacturer’s available catalogs. All that is needed is the beam span, the number of spans and the load value. Cross-section warping and distortions are ignored in this study, although they usually affect the behavior of thin-walled beams. These phenomena are widely discussed in the literature, including buckling assessment of thin-walled steel frames [3], where Generalized Beam Theory (GBT) was used, or nonlinear torsional analysis of composite beams [4], where Benscoter theory was applied. The nonlinear behavior can be also considered similarly to, for example, the approach presented in [5], where non-uniform distortions and warping of the cross-section in the beam model and Saint Venànt solutions were taken into account. A phenomenon not included here, but equally important, is also the influence of torsion and shear distortion of the beam, which were analyzed, among others, by Addessi et al. in [6].

Calculations of geometric or strength parameters for a fixed beam without holes is easy to perform. However, for more complicated structures or elements with holes, more complex calculations are required. In the case of complex cross-section consisting of several materials, the analytical calculations can usually be performed only for the elastic range. The problem arises when one takes into account the plasticity of the material, then the section properties are not sufficient to determine the load capacity of the structure. In this case, the application of general nonlinear constitutive law (GNCL) proves to be useful. Thus far, they have already been used for 2D steel plates [7], composite beams [8], trapezoidal sheets [9] and hollow-core slabs [10]. If the cross-section changes along the length of the beam, additional partitioning and averaging techniques are required for the analytical problem. Sometimes an individual analytical solution is required, typically aimed at a unique cross-section. However, this solution is very labor-intensive and the use of it becomes impractical, since usually an engineering design needs to be obtained quickly and universally. Therefore, the remedy is to use numerical methods, especially the finite element method (FEM), which is currently one of the most widely used method by civil engineers.

Applying the finite element method and creating full three-dimensional models for complex structures is very time-consuming and labor-intensive. In the case of bar elements with perforations or holes, modelling with the use of solid or shell elements is required [1,2]. In this case, the classical FEM becomes ineffective due to the need of dense mesh in the vicinity of the holes. Additionally, shell or solid elements are necessary to be implemented in commercial or home-made finite element method code. Therefore, simplified approaches are often used. An example of such simplification may be the use of one of the homogenization techniques available for FEM calculations. Such an approach could significantly simplify the calculations, thus, reducing time.

The issue of homogenization has been the subject of numerous studies over the last dozen or so years. Consequently, many homogenization techniques have emerged. There are homogenization methods based on the deformation energy [11], the periodic homogenization [12,13] or based on the equivalence of the deformation energy [14,15,16,17,18]. The paper [17] presents a method of determining the stiffness of perforated corrugated board, which is based on the homogenization method presented by Garbowski and Gajewski [15]. It is a universal method used not only for cardboard homogenization, but also for engineering structures. For instance, it was used for the homogenization of the cross-section of prefabricated Filigran slabs in the work of Staszak et al. [16] and for thin-walled profiles with holes [18]. On the other hand, in [19] the method based on the matrix eigenvectors and the main vectors of the state transfer matrix for the lattice beam-like structure was used. The authors of [20] present the homogenization of the three-dimensional model of non-centrosymmetric tetrachiral unit cells based on homogenization in bundles taking into account the Timoshenko theory. The above examples show that the homogenization methods in each case accelerated the numerical calculations or significantly simplified the calculation model.

Thanks to the use of homogenization methods, calculations can be accelerated, which is especially desirable in optimization problems [21,22]. For instance, homogenization was used with great time benefit to obtain the geometrical [23] and material [24] parameters of the structure. The authors of [25] describe the optimization of the cross-section of a thin-walled section of a structure with dynamic behavior. Additionally, in the works [22,26,27], the optimization of the structure topology was presented. In turn, in [28] the optimization of the sigma type cross-section and in [29] the C-type cross-section were presented. In [30], the authors showed the use of Latin hypercube sampling (LHS) for square tube trusses with holes. A hybrid optimization method is presented in [31]. The authors used this technique to optimize cellular beams.

In this paper, we use the homogenization technique based on the equivalence of elastic strain energy and traditional gradient-based minimization algorithms for parametric optimization of thin-walled sections of different beams. After the successful implementation of a very efficient algorithm in MATLAB, an offline computation method based on a surrogate was also presented. Its task was to replace the homogenization procedure, which further accelerated the optimization process without significantly losing the accuracy of the proposed procedure. In addition, a properly prepared surrogate can be used on a small portable computer or as a light and fast web application to optimize any parameterized cross-sections of thin-walled beams with holes; of course, after prior training of the model using the procedure proposed here.

## 2. Materials and Methods

### 2.1. Study Objective and Optimization Frameworks

Optimization problems are common in daily engineering work. Often they are solved intuitively, or by trial and error methods. Highly qualified engineers use advanced optimization methods available in commercial software, developed by in-house codes or possibly implemented as the subroutines/addons to their daily usage programs. One of the key elements is the so-called solver of each optimization task, which must be able to map the output results based on the input parameters.

In advanced engineering problems, the solver is rarely a simple analytical or empirical mathematical expression. Most frequently, the output is obtained by heavy and medium-heavy computations, for instance, by employing the finite element method [32,33], meshless methods [34,35], etc. Therefore, it is greatly desirable to use the numerical techniques which are able to reduce the number of problem degrees of freedom while maintaining the accuracy of modelling. Such property can be achieved by adopting the numerical technique proposed in Staszak et al. [18].

In this paper, the shell-to-beam homogenization technique [18] was used as part of an optimization problem in structural engineering to model the representative volume element (RVE) stiffness of thin-walled 3D perforated beams. The general question considered in the study was: what should a cross-section design of a thin-walled 3D perforated beam from a Z, C or Σ profile be for a simply supported beam with an evenly distributed load to achieve a specific load capacity due to bending moment and maximum displacement. The cross-section design was described by a parametric model of $\bar{x}$; see Section 2.2. Beam length used was equal to 1.0 m and the evenly distributed load was equal to 1 kN/m. The beam was assumed to be rotated in the cross-section with α=25°, therefore, the bending moment and displacement was computed in x and y direction (distributed adequately by trigonometric functions). The resultant bending moment criteria and displacement criteria set was $M_{cr}=2.65\;\mathrm{kNm}$ and $d_{cr}=0.0696\;\mathrm{mm}$ for Z profile, $M_{cr}=2.89\;\mathrm{kNm}$ and $d_{cr}=0.105\;\mathrm{mm}$ for C profile and $M_{cr}=2.21\;\mathrm{kNm}$ and $d_{cr}=0.145\;\mathrm{mm}$ for Σ profile.

The load-bearing bending moment $M\left(\bar{x}\right)$ was computed from ultimate stresses in two-direction bending:

(1) $$\sigma_{pl}=\frac{M_{y}}{I_{y}\left(\bar{x}\right)}x_{max}\left(\bar{x}\right)+\frac{M_{x}}{I_{x}\left(\bar{x}\right)}y_{max}\left(\bar{x}\right),$$

where $\sigma_{pl}$ is the yield stress, here assumed to be 235 MPa; $M_{x}$ and $M_{y}$ are bending moments; $I_{x}$ and $I_{y}$ are moments of inertia along x and y axes, respectively; and $x_{max}$ and $y_{max}$ are the maximal coordinates in the cross-section plane. After substituting $M_{y}=cos\alpha \cdot M\left(\bar{x}\right)$ and $M_{x}=sin\alpha \cdot M\left(\bar{x}\right)$ we can extract the following form of load-bearing bending moment:

(2) $$M\left(\bar{x}\right)=\frac{\sigma_{pl}\cdot I_{x}\left(\bar{x}\right)\cdot I_{y}\left(\bar{x}\right)}{cos\alpha \cdot y_{max}\left(\bar{x}\right)\cdot I_{y}\left(\bar{x}\right)+sin\alpha \cdot x_{max}\left(\bar{x}\right)\cdot I_{x}\left(\bar{x}\right)}.$$

In perforated beams, the displacement from shearing can be the important part of the overall displacements. Therefore, in the study, the second order displacements were also taken into consideration:

(3) $$\begin{array}{ccc} d_{i}\left(\bar{x}\right)=\frac{5q_{i}\cdot L^{4}}{384\cdot EI_{j}\left(\bar{x}\right)}+\frac{q_{i}\cdot L^{2}}{8\cdot G_{jz}A\left(\bar{x}\right)} & i=x,\;y & j=y,x \end{array}.$$

where L is the beam length and q is the evenly distributed load.

As mentioned earlier, the displacements were computed in x and y directions, thus, the resultant values are computed from the simple formula:

(4) $$d\left(\bar{x}\right)=\sqrt{d_{x}{\left(\bar{x}\right)}^{2}+d_{y}{\left(\bar{x}\right)}^{2}}.$$

To sum up, after considering Equations (2) and (4), the objective function, F, used in the study in all optimization jobs has the following form:

(5) $$F\left(\bar{x}\right)=\left|\frac{d\left(\bar{x}\right)-d_{cr}}{d_{cr}}\right|+\left|\frac{M\left(\bar{x}\right)-M_{cr}}{M_{cr}}\right|.$$

**Figure 1:**
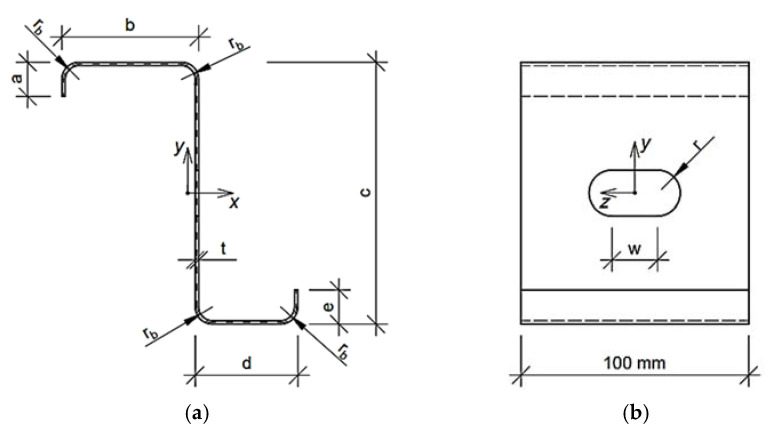
Z profile parametrized for optimization purpose: (**a**) Cross-section and (**b**) side view of representative volume element.

**Figure 2:**
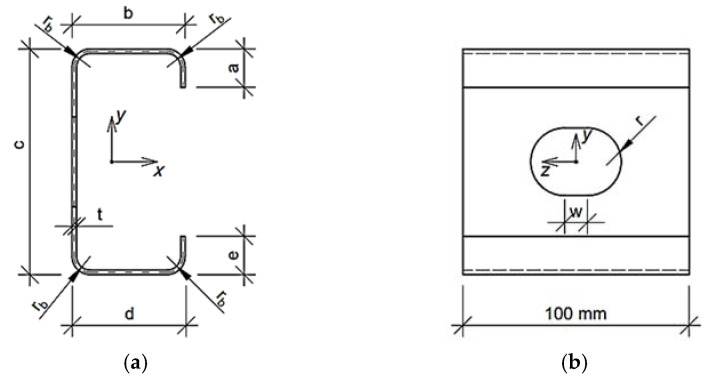
C profile parametrized for optimization purpose: (**a**) Cross-section and (**b**) side view of representative volume element.

**Figure 3:**
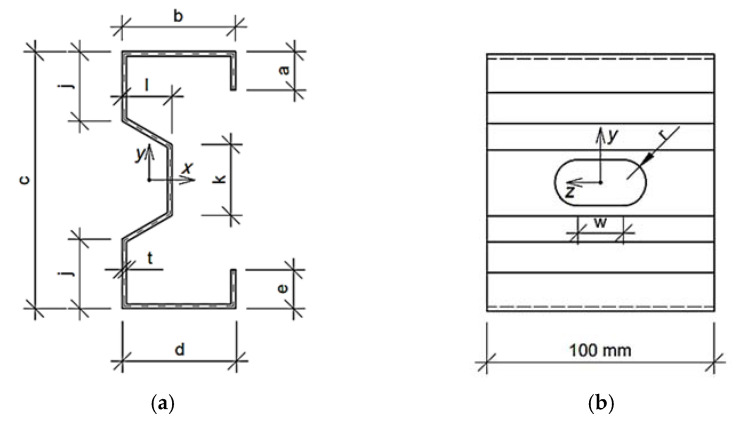
Σ profile parametrized for optimization purpose: (**a**) Cross-section and (**b**) side view of representative volume element.

Such form of Equation (5) enforces the optimization algorithm to find the solution with $M_{cr}$ and $d_{cr}$ assumed earlier in this section. Because we solve the engineering problem the boundary conditions are physical dimensions. Each parameter has lower, $b_{l}$, and upper boundary, $b_{u}$; the boundary conditions are presented in Table 1 (the meaning of the symbols are described at the beginning of Section 2.2 and drawn in Figure 1, Figure 2 and Figure 3). The problem was solved for two fixed levels of thin-walled sheet thicknesses, namely, 1.5 and 2.0 mm. 

The main objective of the paper was to verify different approaches to solve the constrained optimization problem. Three cases were selected, namely, (i) traditional optimization with minimization algorithm of sequential quadratic programming (SQP) [36], (ii) optimization with metamodeling by radial basis function (RBF) network feed with systematic sampling data and (iii) optimization with metamodeling by RBF network feed with optimal Latin hypercube sampling (OLHS) data. In order to find the global solution, the optimization algorithm was run from several initial guesses of design parameters; see Table 2. All computations in the research were performed in MATLAB software (Software Version 2021b, Mathworks, Natick, MA, USA).

### 2.2. Parametric Models of Thin-Walled Cross-Sections

In the study, the three cold formed thin-walled cross-sections were considered, i.e., Z profile, C profile and Σ profile. The Z and C profiles have rounding at the corners and regular holes in the web. The hole shapes considered are circular and stadium type. The geometries analyzed for Z and C profiles were parameterized by a few geometric features:

- b—upper flange width
- c—web height
- d—lower flange width
- t—thin-walled sheet
- w—length of the rectangular part of the hole
- r—radius of the hole

The Σ profile was parametrized by:

- b—upper/lower flange width
- c—overall height
- j—non-perforated height of the web
- k—perforation height
- l—web perforation depth

Moreover, some of the features are dependent on the geometric parameters assumed, that is, a—length of upper flange stiffener is equal to 10t, e—length of lower flange stiffener is equal to 10t, and r_b_—bending radius in the cross-section are equal to 3t. 

The parametrized models were used to build the representative volume element (required in the numerical homogenization technique [18]) for a particular set of parameters used in the optimization framework. The technique is based on the finite element method approach, but does not require formal computational stress analyses, i.e., solving a system of equations.

### 2.3. Numerical Homogenization Technique

Although, in the case of thin-walled beam elements, the material from which they are made is rarely heterogeneous, the appearance of holes along the beam means that traditional tools and algorithms cannot be used. To avoid the need for advanced tools and complicated modeling, numerical homogenization can be used [12,14,15,16,17]. In this study, the homogenization method proposed in [18] was used, which was an adaptation (to thin-walled beams) of the technique based on the elastic deformation energy equivalence [14]. This method was also successfully used earlier for the homogenization of layered sections of shells with a corrugated core [15,17] and concrete slabs reinforced with spatial trusses [16].

In order to apply the elastic deformation energy equivalence between the full 3D model (which is homogenized) and the model reduced to a structural element, the first step is to correctly construct the relationship between the deformation and the displacement in the selected model nodes. To do this, first one needs to build a full stiffness matrix of RVE and then condense the stiffness of the full model into nodes where kinematic boundary conditions have been applied. Let us briefly go through the homogenization method used in [18] and adopted in the study. For more details, the reader should follow [18], in which all mathematical details of the method were stated. 

Starting from a typical finite element method equation, one may extract magnitudes for external (subscript e) and internal (subscript i) nodes:

(6) $$K\;u=F\;\to \left[\begin{array}{cc} K_{ee} & K_{ei}\\ K_{ie} & K_{ii} \end{array}\right]\left[\begin{array}{c} u_{e}\\ u_{i} \end{array}\right]=\left[\begin{array}{c} F_{e}\\ 0 \end{array}\right]\;.$$

where K is the stiffness matrix, **u** is the nodal displacement vector and F is the external nodal load vector. External nodes are the ones at the front and back of the RVE cross-section. The stiffness matrix of external nodes of RVE may be computed from static condensation, i.e.,

(7) $$K_{e}=K_{ee}-K_{ei}\;K_{ii}{}^{-1}\;K_{ie}\;,$$

On the other hand, the total elastic strain energy, E, may be written as:

(8) $$E=\frac{1}{2}\;u_{e}^{T}\;F_{e}\;.$$

Which, after a series of substitutions and simplifications, can be expressed as:

(9) $$E=\frac{1}{2}\varepsilon_{e}^{T}\;H_{k}\;\varepsilon_{e}\left\{length\right\},\;$$

where $H_{k}$ contains compression, bending and shearing stiffnesses:

(10) $$H_{k}=\frac{H_{e}^{T}\;K_{e}\;H_{e}}{\left\{length\right\}}=\left[\begin{array}{ccccc} A_{33} & B_{31} & B_{32} & 0 & 0\\ B_{13} & D_{11} & 0 & 0 & 0\\ B_{23} & 0 & D_{22} & 0 & 0\\ 0 & 0 & 0 & R_{44} & 0\\ 0 & 0 & 0 & 0 & R_{55} \end{array}\right]\;,$$

where: $A_{33}$—tensile stiffness along longitudinal axis; $D_{11}$ and $D_{22}$—bending stiffnesses with respect to the in-plane directions; $R_{44}$ and $R_{55}$—shear stiffnesses of RVE and $B_{13}=B_{31}$ and $B_{23}=B_{32}$—the terms of compressive-bending behavior.

The most important part of the method is the transformation matrix $H_{i}$ for each node ($x_{i}=x$, $y_{i}=y$, $z_{i}=z$), i.e., the approach for coupling nodal displacements and nodal deformation in:

(11) $$u_{i}=H_{i}\;\varepsilon_{i}\;,$$

The full form of Equation (11), in the method postulated [18] is the following:

(12) $${\left[\begin{array}{c} u_{x}\\ u_{y}\\ u_{z}\\ \theta_{x}\\ \theta_{y} \end{array}\right]}_{i}={\left[\begin{array}{ccccc} 0 & 0 & -z^{2}/2 & z/2 & 0\\ 0 & -z^{2}/2 & 0 & 0 & z/2\\ z & yz & xz & x/2 & y/2\\ 0 & 0 & -z & 0 & 0\\ 0 & -z & 0 & 0 & 0 \end{array}\right]}_{i}{\left[\begin{array}{c} \varepsilon_{z}\\ \kappa_{x}\\ \kappa_{y}\\ \gamma_{xz}\\ \gamma_{yz} \end{array}\right]}_{i}.$$

### 2.4. Optimization Methods Used

#### 2.4.1. Sequential Quadratic Programming

Sequential quadratic programming (SQP) method was used because this optimization method is one of the most efficient, accurate and was tested over a large number of benchmarks. Moreover, it has a high percentage level of successful solutions [37,38,39,40,41].

The mathematical problem of the optimization is postulated in the following manner:

(13) $$\min F\left(\bar{x}\right),$$

where $F\left(\bar{x}\right)$ is the objective function of design parameters $\bar{x}$ with potential equalities constraints:

(14) $$\begin{array}{c} C_{eq}\left(\bar{x}\right)=0,\\ A_{eq}\cdot \bar{x}=b_{eq}, \end{array}$$

and inequalities constraints:

(15) $$\begin{array}{c} C\left(\bar{x}\right)\leq 0,\\ A\cdot \bar{x}\leq b,\\ b_{l}\leq \bar{x}\leq b_{u}, \end{array}$$

where b and $b_{eq}$ are vectors; A and $A_{eq}$ are matrices; C and $C_{eq}$ are functions; $b_{l}$ and $b_{u}$ are vectors of lower and upper boundary values of design parameters $\bar{x}$. 

In order to take the constraints into account, the function $F\left(\bar{x}\right)$ is modified according to Lagrange’s function, L, which takes the subproblem form:

(16) $$L\left(\bar{x},\lambda \right)=F\left(\bar{x}\right)+\overset{m}{\underset{i=1}{\sum }}\lambda_{i}\cdot g\left(\bar{x}\right),$$

where $\lambda_{i}$ are the Lagrange multipliers and $g_{i}(\bar{x}$) are the nonequality constraints.

The quadratic programming subproblem form leads to the following expression:

(17) $$\underset{d\in R^{n}}{\min}\frac{1}{2}d^{T}H_{k}+\nabla F{\left({\bar{x}}_{k}\right)}^{T}d,$$

where $H_{k}$ is the positive definite approximation of the Hessian matrix of Equation (16). The approximation is obtained bye Broyden–Fletcher–Goldfarb–Shanno (BFGS) method. The Hessian matrix is updated at each major iteration by the following expression:

(18) $$H_{k+1}=H_{k}+\frac{q_{k}q_{k}^{T}}{q_{k}^{T}s_{k}}-\frac{H_{k}s_{k}s_{k}^{T}H_{k}^{T}}{s_{k}^{T}H_{k}s_{k}},$$

where

(19) $$s_{k}={\bar{x}}_{k+1}-{\bar{x}}_{k},$$

(20) $$q_{k}=\left(\nabla F\left({\bar{x}}_{k+1}\right)+\overset{m}{\underset{i=1}{\sum }}\lambda_{i}\nabla g_{i}\left({\bar{x}}_{k+1}\right)\right)-\left(\nabla F\left({\bar{x}}_{k}\right)+\overset{m}{\underset{i=1}{\sum }}\lambda_{i}\nabla g_{i}\left({\bar{x}}_{k}\right)\right).$$

The solution obtained due to quadratic programing is used to generate a new step:

(21) $${\bar{x}}_{k+1}={\bar{x}}_{k}+\alpha_{k}d_{k},$$

where $\alpha_{k}$ is the step length received to decrease the discrepancy in merit function [38,39,40].

#### 2.4.2. Radial Basis Function Network

In this work, we used very simple neural networks with radial basis functions. These networks are characterized by zero error on the training vectors. The training parameters of such a neural network are: (1) a set of input vectors P (in our case a design parameter of the cross-section $\bar{x}$), (2) target vectors T (stiffnesses calculated through the numerical homogenization) and (3) a certain constant defining spread for the radial layer base.

As a result of learning, we obtain weights and biases in such a way that the outputs of the network are exactly the values of T, when the inputs are the value of P. The network has as many radial neurons as there are input vectors in P and sets the weights of the first layer to the value of P. Thus, there is a layer of radial neurons in which each neuron acts as a detector for a different input vector. The transfer function, a, is the following [42]:

(22) $$a\left(n\right)=\exp\left(-n^{2}\right),$$

where n is a distance between the value of $P_{i}$ and the value of weight $W_{i}$.

Each variation in the first layer is set so that for a radial basis function that intersects 0.5, the weighted inputs value is +/− the spread value. This determines the width of the area in the input space to which each neuron corresponds. Second layer weights and deviations are found by simulating the first layer results a and then solving a simple system of linear equations (for known values of T targets).

Thus, the only required condition in this type of neural network is to ensure that the spread is large enough that the active input regions of the radial neurons overlap sufficiently (to always activate several radial neurons with a given input vector). This makes the approximation function smoother and the results are better generalized for new input vectors located between the input vectors used for training. The spread for each neuron should not be too large to be able to respond effectively to a correspondingly large area of the input space. In our case, the value of this parameter was set to 10; this was the value that generated the smallest error when testing the network with new input vectors.

### 2.5. Submodelling Sampling

#### 2.5.1. Systematic Exploration

In the work, the systematic sampling data was used in (ii) optimization task, assuming the boundary design parameters $\bar{x}$ included in Table 1. For each parameter, a certain number of evenly spaced samples was assumed. These were, respectively, three values for parameters t and w, four values for parameters b, d, r and five values for parameter c. The parameters are shown in Figure 1 and Figure 2. As a result, 2880 sets of design parameters $\bar{x}\;$were obtained within the boundary adopted in Table 1. Then those parameters were used in (ii) optimization task for RBF training for optimization with metamodeling.

##### 2.5.2. Optimal Latin Hypercube Sampling

In the study, the optimal Latin hypercube sampling was used in (iii) optimization job within the assumed boundary of design parameters $\bar{x}$; see Table 1. The optimal Latin hypercube sampling algorithm in its first step spreads the random Latin hypercube design matrix sampling. In the next step, the two factor levels are replaced in the column matrix, which gives new sampling spacing, and this is repeated until the goal is achieved. The goal of the algorithm is to obtain the spread of the points (sampling of the $\bar{x}$ parameters) as evenly as it is possible. 

The algorithm in order to generate the sampling is using the maximum distance criteria, $\phi_{p}$, according to Jin et al. [43]:

(23) $$\phi_{p}={\left(\overset{s}{\underset{i=1}{\sum }}J_{i}d_{i}^{-p}\right)}^{1/p},$$

where p is a positive integer, $J_{i}$ is the index, s is the number of distinct distance values and $d_{i}$ is the distance between two sample points.

In the study, for (iii) optimization job, 75% of the points used for systematic sampling optimization, i.e., (ii) optimization job, was assumed, namely, 2160 points were generated, i.e., set of 2160 design parameters $\bar{x}$ within the boundary assumed in Table 1 were obtained and used in learning a radial basis function network approximator.

### 2.6. Surrogate Model

In the methodologies presented above, the artificial neural network with radial basis functions (see Section 2.4.2) is a surrogate (i.e., a blackbox) that replaces the very laborious process of creating a numerical model and homogenization. In this research work, two models trained a priori, with the use of training data based on a regular grid of points in the parameter space (see Section 2.5.1) and the LHS grid (see Section 2.5.2) are used. Both models prepared once and for all in the learning process can be used offline (without the need to involve specialized numerical analysis software) as a module in an optimization algorithm (see Section 2.4.1).

## 3. Results

The flow of the computations in the optimization is as follows:

- design parameters $\bar{x}$ are passed to the objective function $F\left(\bar{x}\right)$ (due to initial guess or optimization update, Equation (21))
- these design parameters $\bar{x}$ are used to compute, via numerical homogenization, the effective stiffnesses (Equation (10))
- these effective stiffnesses are used in Equations (2) and (3) to compute the objective function values F(x), Equation (5), at each iteration step of the optimization process
- F(x) is minimized according to Equations from (13) to (21).

The sensitivity analysis of objective functions for each parameter for all profiles used were computed. For each trial step in the SQP optimization the numerical gradient of the objective function was computed. These data were used to compute the mean sensitivity for each parameter. For Z and C profiles the results were similar; for b, c, d, w, r the sensitivities for the Z profile were 1.23%, 1.07%, 0.59%, 0.75%, 0.76% and 0.79% and 1.28%, 1.06%, 0.50%, 0.81, 0.82% and 0.93% for the C profile. For b, c, j, k and l for the Σ profile the sensitivities were 5.32%, 1.13%, 1.33%, 1.79% and 1.50%.

### 3.1. Traditional Optimization with SQP Minimization Algorithm

#### 3.1.1. Z Profile

The Z profile results obtained for traditional optimization with SQP minimization algorithm are presented in Table 3. Data show the derived optimal parameters for particular thickness of thin-walled sheet (1.5 mm or 2.0 mm) for each initial guess presented in Table 2. The last column demonstrates the objective function obtained at optimal point derived. For 1.5 mm the lowest value was achieved for the fourth case, i.e., $F^{\left(i\right)}=0.0079$. For 2.0 mm the lowest value was achieved for the fourth case, i.e., $F^{\left(i\right)}=0.0057$. 

**Figure 4:**
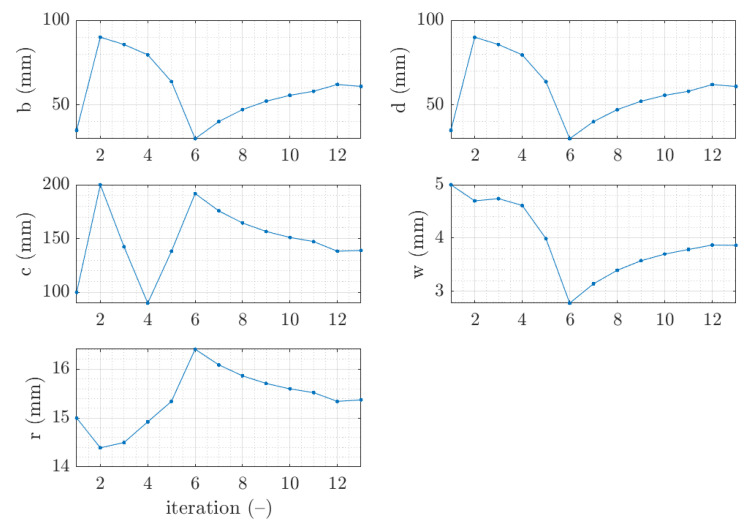
Parameter convergence of Z profile for traditional optimization for ${\bar{x}}_{4}$ initial guess.

**Figure 5:**
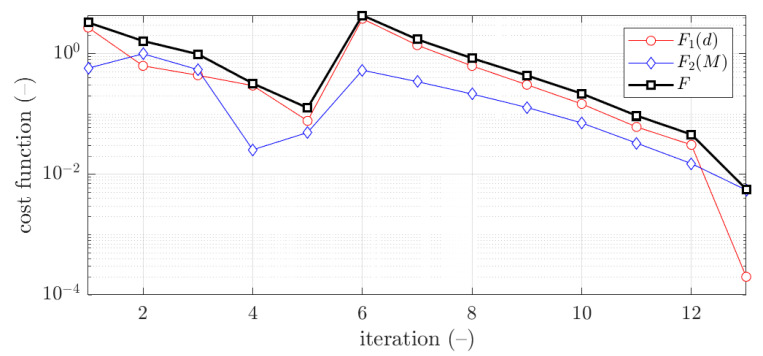
Objective function minimization of Z profile for traditional optimization for ${\bar{x}}_{4}$ initial guess (thickness 2.0 mm ).

The example of the design parameters convergence for the fourth case and 2.0 mm thickness is shown in Figure 4. Moreover, in Figure 5, for the same case the objective function F is illustrated for minimization iterations. The objective function components were also presented; $F_{1}$, which was responsible for deflection discrepancy and $F_{2}$, which accounted for load-bearing moment discrepancy.

#### 3.1.2. C Profile

The C profile results obtained for traditional optimization with SQP minimization algorithm were presented in Table 4. Data show the derived optimal parameters for particular thickness of thin-walled sheet (1.5 mm or 2.0 mm) for each initial guess presented in Table 2. The last column demonstrates the objective function obtained at optimal point derived. For 1.5 mm the lowest value was achieved for the first case, i.e., $F^{\left(i\right)}=0.0095$. For 2.0 mm the lowest value was achieved for the first case, i.e., $F^{\left(i\right)}=0.0073$. 

**Figure 6:**
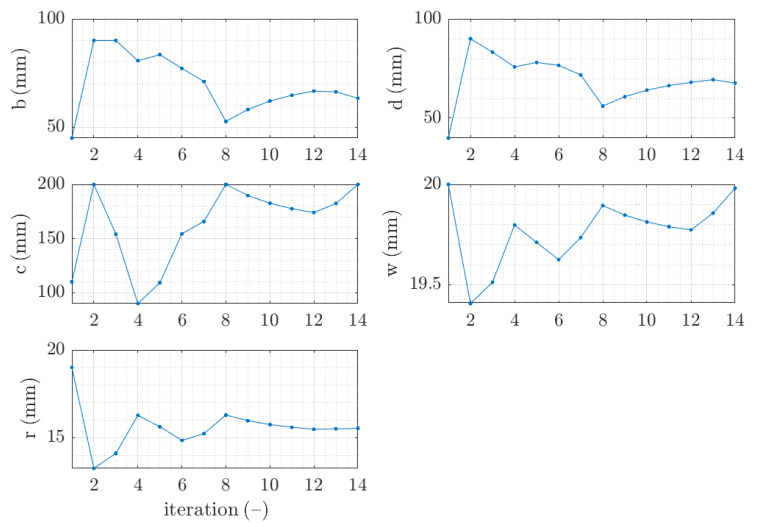
Parameter convergence of C profile for traditional optimization for ${\bar{x}}_{1}$ initial guess.

**Figure 7:**
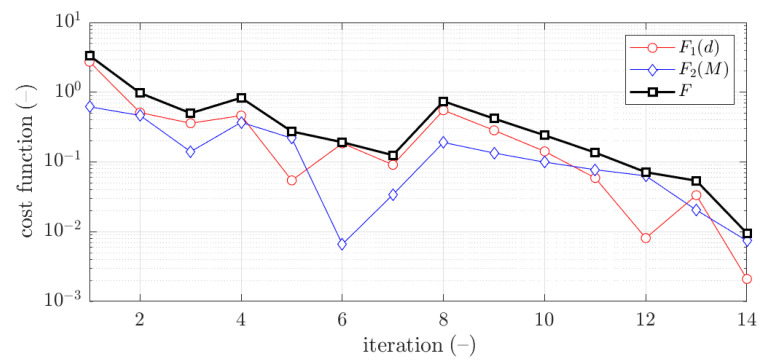
Objective function minimization of C profile for traditional optimization for ${\bar{x}}_{1}$ initial guess (thickness 1.5 mm ).

The example of the design parameters convergence for the first case and 1.5 mm thickness is shown in Figure 6. Moreover, in Figure 7, for the same case the objective function F was illustrated for minimization iterations. As previously, the objective function components are also presented, $F_{1}$ and $F_{2}$.

#### 3.1.3. Σ. Profile

The Σ profile results obtained for traditional optimization with SQP minimization algorithm are presented in Table 5. Data show the derived optimal parameters for particular thickness of thin-walled sheet (1.5 mm or 2.0 mm) for each initial guess presented in Table 2. The last column demonstrates the objective function obtained at optimal point derived. For 1.5 mm the lowest value was achieved for the fourth case, i.e., $F^{\left(i\right)}=0.026$. For 2.0 mm the lowest value was achieved for the second case, i.e., $F^{\left(i\right)}=0.0104$. 

**Figure 8:**
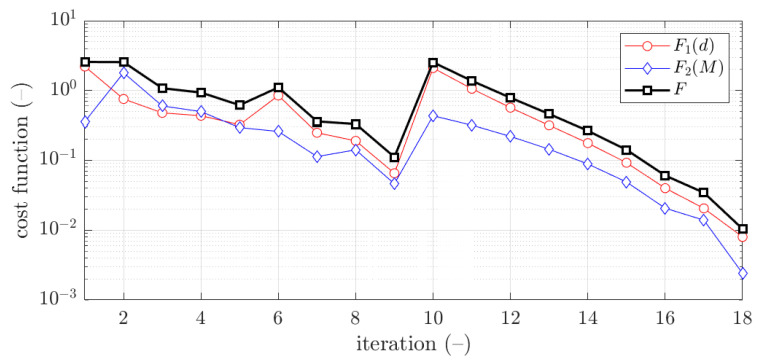
Objective function minimization of Σ profile for traditional optimization for ${\bar{x}}_{2}$ initial guess (thickness 2.0 mm ).

In Figure 8, for the second case the objective function F was illustrated for minimization iterations. As previously, the objective function components are also presented, $F_{1}$ and $F_{2}$.

### 3.2. Optimization with Radial Basis Function Metamodeling Feed with Systematic Sampling Data

#### 3.2.1. Z Profile

The Z profile results obtained for optimization with radial basis function metamodeling feed with systematic sampling data were presented in Table 6. Data show the derived optimal parameters for particular thicknesses of thin-walled sheet (1.5 mm or 2.0 mm) for each initial guess presented in Table 2. The last column demonstrates the objective function obtained at optimal point derived. For 1.5 mm the lowest value was achieved for the fourth case, i.e., $F^{\left(ii\right)}=0.0058$. For 2.0 mm the lowest value was achieved for the fourth case, i.e., $F^{\left(ii\right)}=0.0043$. In brackets, the values from exact RVE modelling were presented. 

**Figure 9:**
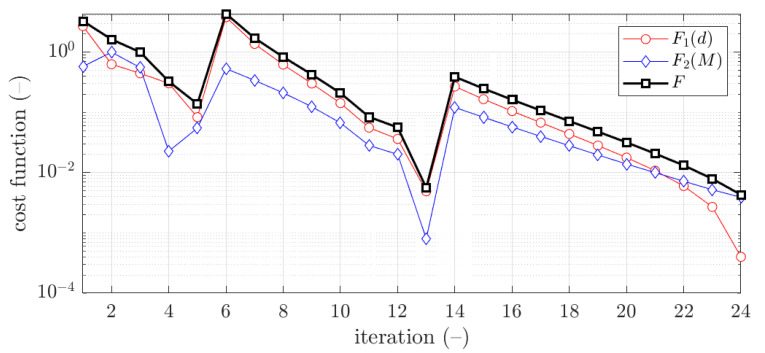
Objective function minimization of Z profile for optimization with metamodeling by RBF adapted feed with systematic sampling data for ${\bar{x}}_{4}$ initial guess (thickness 2.0 mm ).

The example of the objective function convergence F for fourth case and 2.0 mm thickness is shown in Figure 9. As previously, the objective function components are also presented, $F_{1}$ and $F_{2}$.

#### 3.2.2. C Profile

The C profile results obtained for optimization with radial basis function metamodeling feed with systematic sampling data are presented in Table 7. Data show the derived optimal parameters for particular thicknesses of thin-walled sheet (1.5 mm or 2.0 mm) for each initial guess presented in Table 2. The last column demonstrates the objective function obtained at optimal point derived. For 1.5 mm the lowest value was achieved for the fifth case, i.e., $F^{\left(ii\right)}=0.0091$. For 2.0 mm the lowest value was achieved for the first case, i.e., $F^{\left(ii\right)}=0.0024$. In brackets, the values from exact RVE modelling are presented. 

**Figure 10:**
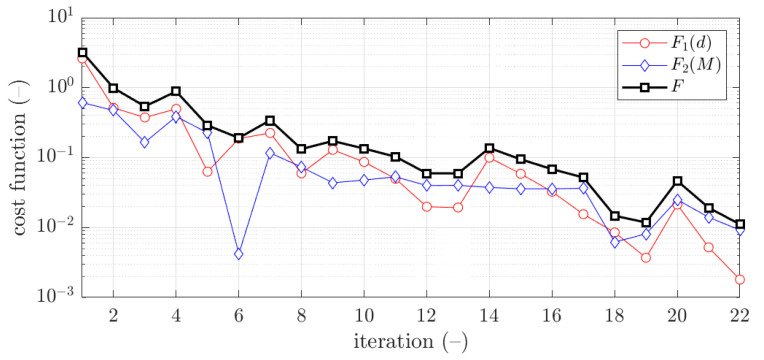
Objective function minimization of C profile for optimization with metamodeling by RBF adapted feed with systematic sampling data for ${\bar{x}}_{1}$ initial guess (thickness 1.5 mm ).

The example of the objective function convergence F for first case and 1.5 mm thickness is shown in Figure 10. As previously, the objective function components are also presented, $F_{1}$ and $F_{2}$.

### 3.3. Optimization with Radial Basis Function Metamodeling Feed with Optimal Latin Hypercube Sampling Data

#### 3.3.1. Z Profile

The Z profile results obtained for optimization with radial basis function metamodeling feed with optimal Latin hypercube sampling data are presented in Table 8. Data show the derived optimal parameters for particular thicknesses of thin-walled sheet (1.5 mm or 2.0 mm) for each initial guess presented in Table 2. The last column demonstrates the objective function obtained at optimal point derived. For 1.5 mm the lowest value was achieved for the second case, i.e., $F^{\left(iii\right)}=0.0058$. For 2.0 mm the lowest value was achieved for the fifth case, i.e., $F^{\left(iii\right)}=0.0072$. In brackets, the values from exact RVE modelling are presented.

**Figure 11:**
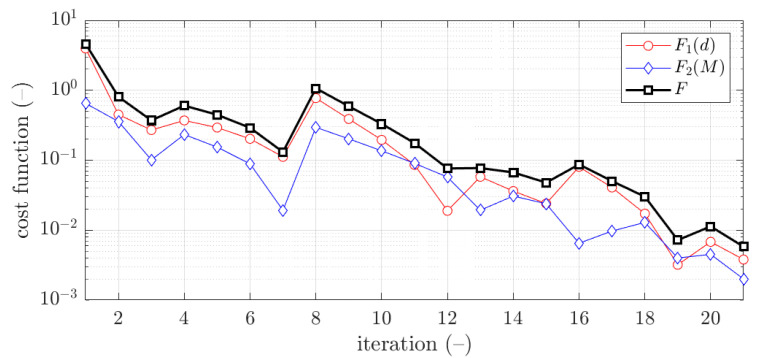
Objective function minimization of Z profile for optimization with metamodeling by RBF adapted feed with OLHS data for ${\bar{x}}_{2}$ initial guess (thickness 1.5 mm ).

The example of the objective function convergence F for second case and 1.5 mm thickness is shown in Figure 11. As previously, the objective function components are also presented, $F_{1}$ and $F_{2}$.

#### 3.3.2. C Profile

The C profile results obtained for optimization with radial basis function metamodeling feed with optimal Latin hypercube sampling data are presented in Table 9. Data show the derived optimal parameters for particular thicknesses of thin-walled sheet (1.5 mm or 2.0 mm) for each initial guess presented in Table 2. The last column demonstrates the objective function obtained at optimal point derived. For 1.5 mm the lowest value was achieved for the first case, i.e., $F^{\left(iii\right)}=0.0196$. For 2.0 mm the lowest value was achieved for the fifth case, i.e., $F^{\left(iii\right)}=0.0029$. In brackets, the values from exact RVE modelling are presented.

**Figure 12:**
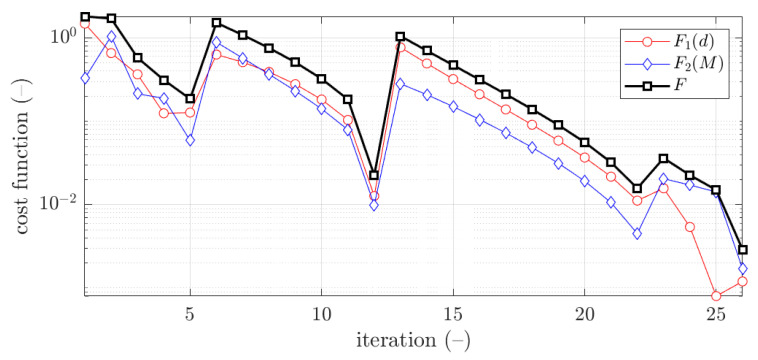
Objective function minimization of C profile for optimization with metamodeling by RBF adapted feed with OLHS data for ${\bar{x}}_{5}$ initial guess (thickness 2.0 mm ).

The example of the objective function convergence F for first case and 2.0 mm thickness is shown in Figure 12. As previously, the objective function components are also presented, $F_{1}$ and $F_{2}$.

## 4. Discussion

The optimization results were presented for three types of optimization tasks: (i) traditional optimization with minimization algorithm of SQP, (ii) optimization with metamodeling by RBF network feed with systematic sampling data and (iii) optimization with metamodeling by RBF network feed with OLHS data. 

The vast majority of optimization tasks have shown good convergence to find optimal solutions and reached the objective function of less than 0.05; in many cases it was less than 0.01, see last column in Table 3, Table 4, Table 5, Table 6, Table 7, Table 8 and Table 9. In traditional optimization (i), for examples presented in Figure 5 and Figure 7, the objective function was gradually decreased, with single modifications during minimizations (in the 6th iteration in Figure 5, and in the 4th and 8th in Figure 7). In RBF optimization with systematic sampling, for examples presented in Figure 9 and Figure 10, the objective function was decreased with some more significant fluctuations (especially in Figure 10) with almost twice the larger number of iterations, i.e., 24 for Z profile and 22 for C profile, respectively. The characteristic rapid increases were observed in Figure 9, in the 6th and 14th iterations, similar issues were not so evident in Figure 10. In RBF optimizations with optimal Latin hypercube sampling, for examples presented in Figure 11 and Figure 12, the objective functions were minimized successfully, in both cases there were more than 20 iterations to find the optimum. In Figure 11, the only rapid increase of the objective function was observed (8th iteration), while three rapid increases were achieved in Figure 12. It is worth noting that the graphs of the objective functions have a logarithmic vertical axis, therefore, the decreases in the last part of the graphs are very small in each case. 

In most cases, the deflection component in the objective function is greater than bending moment component. This feature is clearly visible in Figure 5 and Figure 9, but in both cases, for the optimal solution shown at the end of the graphs, this relationship is inverse, i.e., the bending moment component is greater than the deflection component.

**Figure 13:**
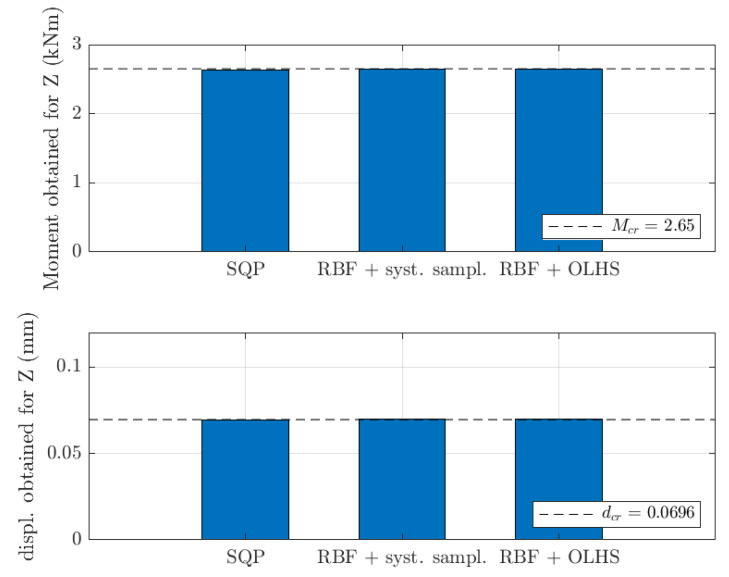
Load-bearing moments and displacements obtained for optimal values of design parameters for Z profile (1.5 mm thickness) for different optimization methods considered in the study.

**Figure 14:**
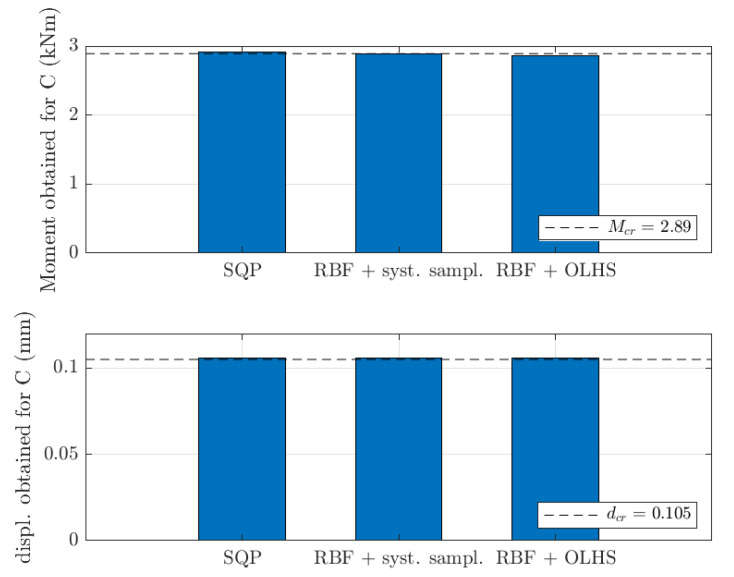
Load-bearing moments and displacements obtained for optimal values of design parameters for C profile (1.5 mm thickness) for different optimization methods considered in the study.

In Figure 13 and Figure 14, the results were summarized for all optimization methods used in the paper, but only for 1.5 mm thickness cases. The values of load-bearing moments and displacements for optimal values of design parameters were presented by bar plots in reference to its sought values (dashed lines). For all optimization cases, very similar magnitudes were achieved to its reference counterparts, i.e., $M_{cr}$ and $d_{cr}$.

Moreover, the optimizations with submodelling (surrogate) approach used have shown a great reduction in computational cost. In optimization (i), the time of computation of a single objective function was about 4.1 s, while in optimization (ii) and optimization (iii) the time was about 0.11 s, i.e., approximately 37 times shorter, which greatly reduced the overall time of computations in those cases. Such time reduction was obtained due to mixing the homogenization technique with soft computing methods applied in optimization. Such a great advantage has not been obtained at the expense of lowering the accuracy of the solution, which was proved by the examples presented; see Section 3.2 and Section 3.3. 

The differences between surrogate models and the exact solutions from the optimal design parameters are show in Table 6, Table 7, Table 8 and Table 9 (last column; the value in the bracket is from the surrogate model, while the values in square brackets comes from the exact solution, i.e., homogenization technique). The differences were small, usually within the range of the optimal solutions derived. For instance, in Table 9, in the first row we have 0.0196, which was obtained for RBF with OLHS, while 0.0186 was the exact value. 

## 5. Conclusions

This paper presents a very efficient algorithm for parametric optimization of thin-walled beam sections. The beams analyzed have various openings along their length and are under various loading and boundary conditions. The proposed procedure uses numerical homogenization based on equivalence of the elastic strain energy between the RVE model and the simplified, structural model. RVE was modeled within the FEM framework as a detailed 3D shell structure and then homogenized to obtain its flexural and shear stiffnesses.

In order to validate the proposed method, the optimization by sequential quadratic programming was performed in the first case. In the next cases, two surrogate models were prepared based on artificial neural networks, i.e., with systematic training set and taken from the optimal Latin hypercube technique. Both allow use of the proposed procedure offline—without the need for numerical modeling. In the online stage, each type of analyzed cross-section was separately recalculated in different geometrical variants and all flexural and shear stiffnesses were calculated by means of shell-to-beam numerical homogenization. Then, on the basis of the systematic training set as well as the ones generated by the optimal Latin hypercube technique, various neural networks with radial base functions were trained for a different type of the beam thin-walled section.

In each case, very precise results, comparable to the reference results, were obtained. This proves the possibility of using artificial neural network models in an offline phase to quickly optimize the cross-sections of various beams with periodic openings under various boundary and load conditions.

## Figures and Tables

**Table 1:** The lower and upper limits of the parameters selected for optimization for Z, C and Σ profiles.

Boundary	Profile	b (mm)	d (mm)	c (mm)	w (mm)	r (mm)
$b_{l}$	Z, C	30	30	90	0	5
$b_{u}$		90	90	200	24	20
**Boundary**	**Profile**	**b and d** **(mm)**	**c** **(mm)**	**j** **(mm)**	**k** **(mm)**	**l** **(mm)**
$b_{l}$	Σ	30	90	20	20	5
$b_{u}$		90	200	45	80	45

**Table 2:** The initial guesses of design parameters selected for optimization for Z, C and Σ profiles.

No.	Profile	b (mm)	d (mm)	c (mm)	w (mm)	r (mm)
${\bar{x}}_{1}$	Z, C	45	40	110	20	19
${\bar{x}}_{2}$	Z, C	35	35	140	5	5
${\bar{x}}_{3}$	Z, C	30	65	90	5	15
${\bar{x}}_{4}$	Z, C	35	35	100	5	15
${\bar{x}}_{5}$	Z, C	45	30	150	10	5
**No.**	**Profile**	**b and d** **(mm)**	**c** **(mm)**	**j** **(mm)**	**k** **(mm)**	**l** **(mm)**
${\bar{x}}_{1}$	Σ	80	150	37.5	50	50
${\bar{x}}_{2}$	Σ	30	120	20	40	20
${\bar{x}}_{3}$	Σ	60	110	37.5	20	35
${\bar{x}}_{4}$	Σ	45	170	20	20	10
${\bar{x}}_{5}$	Σ	55	135	30	30	30

**Table 3:** Results for traditional optimization with SQP minimization algorithm for Z profile.

No.	t (mm)	b (mm)	d (mm)	c (mm)	w (mm)	r (mm)	$F^{\left(i\right)}\;\;\left(-\right)$
1	1.5	82.97	84.08	124.01	19.64	19.16	0.0085
2		87.92	87.92	113.67	4.82	5.06	0.0084
3		63.56	69.52	200	4.53	12.69	0.0554
4		85.14	85.14	119.31	5.6	16.38	0.0079
5		90.0	89.6	112.42	9.19	5.26	0.0259
1	2.0	60.8	57.02	155.88	19.92	18.23	0.0218
2		90.0	90.0	200.0	4.98	5.02	1.6343
3		55.78	62.78	149.35	4.62	13.55	0.0068
4		60.9	60.87	138.94	3.86	15.37	0.0057
5		78.85	48.76	122.68	9.84	6.2	0.0058

**Table 4:** Results for traditional optimization with SQP minimization algorithm for C profile.

No.	t (mm)	b (mm)	d (mm)	c (mm)	w (mm)	r (mm)	$F^{\left(i\right)}\;\;\left(-\right)$
1	1.5	63.37	67.64	200	19.98	15.54	0.0095
2		89.45	89.15	96.69	5.22	5.32	0.1845
3		43.47	88.0	130.68	4.85	14.33	0.0410
4		64.77	65.81	200	5.67	14.85	0.0107
5		85.14	50.86	129.97	9.16	5.26	0.1632
1	2.0	61.26	60.1	145.55	20.17	16.73	0.0073
2		84.92	85.13	90	4.19	6.04	0.1345
3		90	54.49	95.13	4.8	14.14	0.1093
4		86.09	85.94	90	1.64	8.73	0.1341
5		58.09	59.77	144.87	11.07	5.24	0.0112

**Table 5:** Results for traditional optimization with SQP minimization algorithm for Σ profile.

No.	t (mm)	b and d (mm)	c (mm)	j (mm)	k (mm)	l (mm)	$F^{\left(i\right)}\;\;\left(-\right)$
1	1.5	64.51	90	34.23	45.87	45.0	0.1004
2		66.94	90	40.06	20.06	45.0	0.1104
3		54.72	159.5	41.62	22.65	40.5	0.0733
4		56.40	145.1	26.02	20.0	5.0	0.0260
5		54.79	157.6	36.0	31.42	41.87	0.0719
1	2.0	51.82	90.0	30.0	45.6	38.26	0.0280
2		50.49	90.0	43.35	20.0	41.39	0.0104
3		53.48	90.0	35.70	20.0	34.98	0.0237
4		51.77	107.5	31.26	20.77	20.95	0.0491
5		53.43	90.0	35.71	32.40	30.48	0.0241

**Table 6:** Results for optimization with metamodeling by RBF adapted feed with systematic sampling data for Z profile.

No.	t (mm)	b (mm)	d (mm)	c (mm)	w (mm)	r (mm)	$F^{\left(\mathrm{ii}\right)}\;\;\left(-\right)$
1	1.5	83.36	76.4	137.39	20.07	18.55	0.0902
2		87.56	87.55	114.15	4.74	7.09	0.0078
3		58.31	74.06	200	4.55	13.19	0.0531
4		86.46	86.45	117.42	4.96	15.89	0.0058 (0.0248)
5		90	83.52	115.2	9.91	6.44	0.0105
1	2.0	61.6	59.43	142.02	19.68	17.15	0.009
2		66.6	66.55	117.29	5.14	6.98	0.0073
3		60.69	57.7	152.2	4.62	13.33	0.0092
4		60.7	60.7	140.52	5.04	15.82	0.0043 (0.0315)
5		64.71	58.91	132.61	10.15	6.5	0.0129

**Table 7:** Results for optimization with metamodeling by RBF adapted feed with systematic sampling data for C profile.

No.	t (mm)	b (mm)	d (mm)	c (mm)	w (mm)	r (mm)	$F^{\left(\mathrm{ii}\right)}\;\;\left(-\right)$
1	1.5	67.14	63.35	200	19.89	12.23	0.0111
2		90	90	98.89	3.95	14.14	0.1822
3		40.7	80.65	167	4.52	13.9	0.0697
4		69.52	68.63	158.7	3.61	18.29	0.1079
5		83.8	33.78	157.93	10.48	14.52	0.0091(0.0144)
1	2.0	51.68	67.4	144.37	19.93	15.51	0.0024 (0.0030)
2		81.11	79.27	90	6	14.41	0.1109
3		90	54.99	95.02	4.81	14.31	0.1126
4		90	78.81	90	10.53	20	0.0904
5		64.53	53.92	145.78	10.12	10.83	0.0056

**Table 8:** Results for optimization with metamodeling by RBF adapted feed with optimal Latin hypercube sampling data for Z profile.

No.	t (mm)	b (mm)	d (mm)	c (mm)	w (mm)	r (mm)	$F^{\left(\mathrm{iii}\right)}\;\;\left(-\right)$
1	1.5	66.26	67.87	188.8	21.01	17.16	0.0772
2		87.48	87.29	114.64	4.95	5.79	0.0058 (0.0004)
3		56.16	75.61	200	4.4	12.17	0.0567
4		82.95	80.55	122.36	5.61	15.07	0.0484
5		90.0	85.63	113.75	10.7	5.0	0.0113
1	2.0	62.29	56.38	151.84	19.98	17.8	0.0154
2		66.76	67.17	116.47	4.84	5.02	0.0084
3		54.86	62.04	160.03	4.39	12.22	0.0221
4		60.67	61.12	138.63	4.94	14.85	0.0098
5		82.96	32.9	138.04	9.98	8.56	0.0072 (0.0069)

**Table 9:** Results for optimization with metamodeling by RBF adapted feed with optimal Latin hypercube sampling data for C profile.

No.	t (mm)	b (mm)	d (mm)	c (mm)	w (mm)	r (mm)	$F^{\left(\mathrm{ii}\right)}\;\;\left(-\right)$
1	1.5	66.85	64.74	192.81	20.61	14.46	0.0196 (0.0186)
2		89.8	87.98	97.81	4.78	5.57	0.1855
3		40.78	80.43	169.31	4.52	11.2	0.0546
4		75.01	51.8	184.13	3.76	7.68	0.0224
5		88.62	68.07	109.38	10.24	5.42	0.1993
1	2.0	49.86	69.02	142.49	20.26	17.13	0.0163
2		63.0	90.0	90.0	5.92	7.39	0.1053
3		90.0.	53.76	95.26	4.7	13.38	0.1098
4		90.0	90.0	90.0	24.0	20.0	0.0603
5		59.61	59.32	144.03	9.77	7.54	0.0029 (0.0011)

## Data Availability

The data presented in this study are available on request from the corresponding author.
